# Prevalence of symptoms of body dysmorphic disorder (BDD) and associated features in Swiss military recruits: a self-report survey

**DOI:** 10.1186/s12888-021-03288-x

**Published:** 2021-06-07

**Authors:** Marie Drüge, Gabriela Rafique, Anne Jäger, Birgit Watzke

**Affiliations:** Department of Psychology, Clinical Psychology and Psychotherapy Research, University of Zurich, Binzmuehlestrasse 14, 8050 Zurich, Switzerland

**Keywords:** Body dysmorphic disorder, Military medicine, Muscle dysmorphia

## Abstract

**Background:**

Body dysmorphic disorder (BDD), defined as the obsessive idea that some aspect of one’s own body or appearance is severely flawed/deformed, is relatively common in the general population and has been shown to have strong associations with mood and anxiety disorders and substance abuse disorders. Furthermore, a previous study on symptoms of BDD among people in the military showed that muscles are an important area of preoccupation. Hence, this study aimed to 1. assess the prevalence of BDD symptoms in Swiss military recruits, 2. specify the areas of preoccupation, and 3. analyze associated features (depression and alcohol/drug abuse).

**Method:**

A total of 126 Swiss male military recruits (age: M = 20.12, SD = 1.09, range: 18–24) were examined using self-report measurements to assess symptoms of BDD, depression, alcohol/drug abuse.

**Results:**

The results showed that symptoms of BDD were relatively common (9.5% reached the cutoff value for probable BDD, 84% reported some symptoms), with the muscles as the most common area of preoccupation. A positive correlation (r = .38, *p* < .001) between depressive symptoms and symptoms of BDD was found, thus no correlation between alcohol/drug abuse and symptoms of BDD.

**Conclusion:**

The results indicate a need to develop and implement measures for prevention (e.g. raising awareness among the military) and intervention in this specific population.

## Background

Body dissatisfaction or a critical preoccupations with one’s own appearance (e.g. muscles) are considered normal to some extent, but when unwanted thoughts, i.e. intrusions, become too excessive or repetitive behavior becomes time-consuming and causes major distress, the diagnosis of body dysmorphic disorder (BDD) needs to be considered. As BDD onset typically occurs during adolescence, it is vital to identify vulnerable populations or settings with a higher prevalence or risk factors at a younger age in order to tailor specific screenings and/or interventions. In some countries military service including an initial physical examination is mandatory for all young men. Thus, the military might strengthen the focus on physical fitness and increase the vulnerability for being preoccupied with body features, especially with one’s owns muscles, making military personnel prone to a special subtype of BDD, the so-called muscle dysmorphia. To date, there is little research on BDD in the military context, therefore the prevalence of symptoms of BDD and associated factors in this specific population is of interest in the following study.

### Body dysmorphic disorder

Preoccupation with the appearance is relatively common: In a representative sample of 2552 participants of the general population, 27% percent of the males and 41% of the females reported being preoccupied with the appearance of at least one body part [1], without meeting all criteria of body dysmorphic disorder (BDD). According to the DSM-5 [2] BDD, describes the preoccupation with one or more «defects or flaws» of one’s own body part(s) or appearance, which are not apparent to others. This perception leads to repetitive and time-consuming behaviors (such as mirror checking, excessive exercising) or mental acts (such as comparing oneself to others) of some sort. These preoccupations may cause severe educational or occupational dysfunction or social isolation but do not meet the diagnostic criteria of an eating disorder. In the DSM-5, BDD is categorized in the obsessive-compulsive-spectrum [2]. BDD is highly associated with comorbidities such as mood or anxiety disorders [3], and it is associated with a high burden of disease such as impaired psychosocial functioning or high suicide risk. In a prospective study of up to 4 years on 185 patients with BDD, Phillips and Menard [4] analyzed suicide risk among individuals with BDD: 57.8% of respondents reported suicidal ideation, and 2.6% attempted suicide within one year. Two patients died by suicide throughout the study [4].

### Muscle dysmorphia

In the DSM 5 there are two specifiers for BDD: One is with or without muscle dysmorphia, the other differs with or without insight. In individuals with muscle dysmorphia the preoccupation is focused on the muscles or the body built [2]. The idea that their body build is too small or insufficiently muscular might lead towards specific dieting (e. g. proteins), or physical activities (e. g. weightlifting) to increase size and definition of the muscles. Also the preoccupations may cause avoidance (e.g. avoiding situations where the body is exposed) or safety behaviors (e.g. giving up social activities to continue with time-consuming work-out). First described in 1993, Pope et al. [5] conducted a survey to analyze the body images of 108 male body builders; they did not examine BDD or muscle dysmorphia but found something described as a “reverse anorexia” syndrome in nine (8.3%) of the subjects, where the body builders believed that they appeared small and weak even though they were actually tall and muscular. This could be interpreted as a form of muscle dysmorphia.

### Point prevalence

BDD has prevalence rates ranging from 1.7–2.9% [1, 6] and an overall weighted prevalence in the community of 1.9% [7] . As a systematic review found slightly higher prevalences for men than for women [7], some found them to be similar (2.4% vs. 2.2%), but gender differences in the areas of preoccupation have been found (e.g., muscle dysmorphia occurs almost exclusively in males) [8]. Prevalences vary in different samples, such as in student populations (3.3%) or in psychiatric outpatients (5,8%), and the prevalence reached up to 20.1% among patients undergoing rhinoplasty surgery [7]. Point prevalences for muscle dysmorphia are also higher in professional male weightlifters [5, 9].

### Comorbidity

Gunstad and Phillips [3] gave an overview of the lifetime axis I comorbidity rates in published studies of BDD; the most common comorbidities using Structured Clinical Interviews for DSM-III-R (SCID-P) were major depression (range: 8–82%), obsessive compulsive disorder (OCD; range: 6–78%), social phobia (range: 12–69%), and substance use disorders (range: 21–36%). Against the background of the range of comorbidities, they examined comorbidities in 293 patients with BDD; the most common comorbidity was major depression (75.5%), followed by social phobia (36.5%), OCD (32.1%) and substance use disorders (alcohol: 20.5%; other drugs: 17.1%). Furthermore, a correlation between the number of comorbid disorders and the functional impairment of a patient was found.

### Gender differences in BDD

Phillips, Menard and Fay [8] analyzed similarities and differences between 63 men and 137 women suffering from BDD. Though their findings lack generalizability (e.g. due to recruitment of sample limited to northeastern United States), the results are still noteworthy. Men suffering from BDD were significantly older, more likely to be single, and more likely to have their own household than women. The age at onset is within adolescence, thus men had an older age at onset than women (*M* = 17.9, *SD* = 6.9 vs. *M* = 15.9, *SD* = 7.1). Compared to women, the areas of preoccupation among men were more often their genitals (17.5%), body build (36.5%), and thinning hair/balding (36.5%). Men were also obsessed with their jaw (17.5%), nose (38.1%), skin (69.8%), belly (19.0%), and eyes (19.0%), but no differences were found for these areas between men and women [8]. Men were also more likely to have a comorbid substance use disorder than women [8]. Nevertheless, there are few studies focusing on male samples or gender-related research questions.

### Symptoms of BDD in the military

The military requires physical and mental fitness, and pursuing a military career may lead to a rather dysfunctional attention to physical fitness. As mentioned above, if preoccupations about the body build being too small or insufficiently muscular lead through specific behavior patterns to distress and impairment, BDD might occur. Also, the age at BDD onset for men (*M* = 17.9, *SD* = 6.9) is around the period when the initial test of fitness for the military service takes place. Thus, Campagna and Bowsher [10] conducted a survey to determine the prevalence of BDD and muscle dysmorphia in enlisted U.S. military personnel. A total of 13% of male and 21.7% of female participants reported body dysmorphic symptoms in this specific sample [8]. Further analysis showed muscle dysmorphia in 12.7% of the males and 4.2% of the females. As this first study only used self-report measure, and given the complexity to distinguish between body dissatisfaction, eating disorders and BDD, the results should be interpreted with caution. The measures used are only screening tools, and shouldn’t be interpreted as prevalence rates of BDD or MD. Still, it shows, that in this population the preoccupations about body dissatisfaction might be high and focused on the body built or muscular size, especially for men. Recent research on the prevalence of BDD and associated features (e.g., depression) lacks in specific samples (e.g. specific professions), which would be necessary to identify settings and populations where there is a high risk of BDD. This study aims to 1. assess the prevalence of BDD symptoms in Swiss military recruits, 2. specify the areas of preoccupation with particular regard to muscle dysmorphia in this sample, and 3. analyze associated features, such as depressive symptoms and alcohol/drug abuse. Due to feasibility, within the probable associated features the focus was set on depressive symptoms and alcohol/drug abuse.

## Method

### Procedure and sample

We conducted a cross-sectional study aiming at a full sample of recruits of one cohort (company) at the recruit school in Chur, Switzerland. This company of infantry entered the school in 2017, and it was their first year of training when the assessment took place. The recruits were asked to participate voluntarily. A paper-pencil questionnaire was handed out to all recruits on the same day at the recruit school. All recruits participated. The nonclinical sample consisted of 126 male Swiss military recruits (age: M = 20.12 years, SD = 1.09; range: 18–24). Most of the recruits reported being single (74.6%), some of the recruits were in a relationship (23.0%), and two were married (1.6%). Regarding educational level, 69.8% had completed compulsory education, 15.1% had a vocational baccalaureate, 14.3% had baccalaureate, and one recruit had finished a university degree. All recruits had to pass a test on fitness before they began their military service, indicating that from a medical point of view, the recruits were mentally, intellectually and physically fit for service.

### Instruments

In the current study, the prevalence of symptoms of BDD and associated features such as depression, alcohol abuse, and drug abuse were examined using self-reports in a nonclinical, representative sample of Swiss military recruits. To measure BDD, we used a standardized self-report questionnaire for BDD symptoms (Fragebogen Körperdysmorpher Symptome, FKS, [11]). The FKS contains 17 items scored on a 5-point Likert scale (0–4) and one open-ended question to specify the body part (the cutoff value of 14 is used to discriminate BDD; this cutoff has a high sensitivity of 0,87 and a high specificity of 0,93,^1^ α = .88). To measure associated features, we applied standardized self-report questionnaires for depression, including the Allgemeine Depressionsskala (ADS [12];, the German version of the Center for Epidemiological Studies Depression Scale (CES-D; originally published by Radloff [13]), which includes 20 item-scores utilizing a Likert scale (cutoff = 23, α = .82, high specificity). To measure alcohol abuse, we applied the Alcohol Use Disorders Identification Unit (AUDIT [14];), which contains 10 items scored on a 5-point Likert scale (0–4) (cutoff = 15–20; α = .83). To measure drug abuse, we used a single dichotomous question [15]. In the current sample, the internal consistencies were good for FKS (α = .82) and for ADS (α = .84), and acceptable for AUDIT (α = .74).

### Data analysis

The collected data were analyzed using descriptive statistics and correlations (Spearman rank correlation) to analyze links between BDD and associated features. For categorical data, crosstabulations and chi-square tests were used to analyze relations between variables (BDD x associated features) based on the cutoff values.

## Results

### Prevalence of symptoms of BDD

Twelve recruits (9.5%) reached the cutoff value of 14 for the FKS, which indicates the presence of probable body dysmorphic disorder with high sensitivity and specificity. Of these twelve recruits, ten reported being single, and two were in a relationship. Eleven recruits were living with their parents, and one was living alone. Seven of the recruits were born as an only child, the other five were born as the last of two or three children. Regarding educational level, eight recruits had completed compulsory education, two had obtained a vocational baccalaureate, and two had finished a baccalaureate program. Six recruits reported, that they had undergone plastic surgery. Furthermore, 106 recruits (84%) showed some symptoms of BDD: 22 recruits (17.5%) scored 10–13 points, 36 (28.6%) scored 5–9 points, and 49 recruits (38.9%) 1–4 points.

### Specifying the areas of preoccupation, specifically muscle dysmorphia

**Fig. 1:**
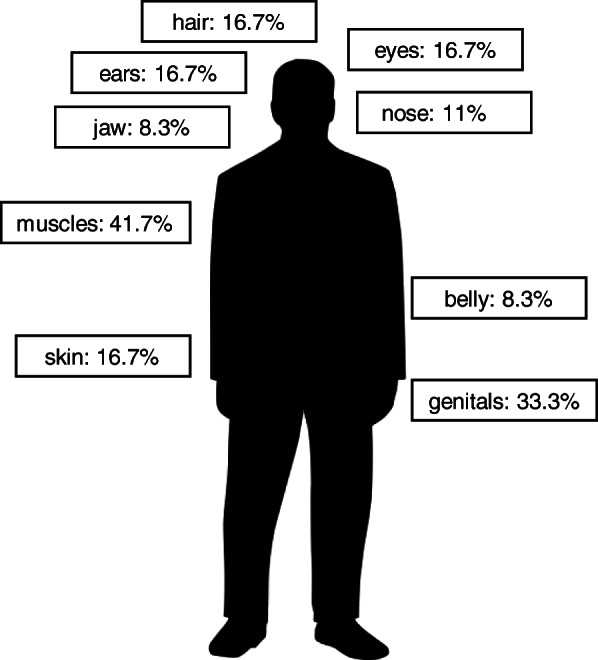
Areas of preoccupation among the recruits with BDD

Taking the area of preoccupation into account, five of the twelve recruits with probable BDD specified their muscles as the area of preoccupation (see Fig. 1); four of the twelve reported their genitals; two their hair, eyes, ears, nose and/or skin; and two mentioned being concerned with their belly and/or the jaw. Five of the twelve recruits reported being preoccupied with only one body part, another five recruits stated two body parts, and two recruits reported three body parts.

### Analyzing associated features

Nine recruits reached the cutoff-score for the ADS, indicating a probable depression with a high specificity. In general, the prevalence of symptoms of BDD correlates positively with depressive symptoms (r = .38, *p* < .01). Furthermore, the results of the AUDIT indicated hazardous or harmful alcohol use among 44 recruits (35.5%). A total of 106 (83.5%) reported the potential for a hazardous use. Fifty-seven recruits (44.88%) had taken an illegal drug at least once, and 38 (29.9%) had taken drugs more than once. No significant relationships between those who reached the cut-off scores for BDD and substance abuse were found.

## Discussion

Our study shows that self-reported symptoms of BDD are common in military recruits. The prevalence of self reported probable BDD herein (9.5%) is substantially higher than the point prevalence of 1.7–2.9% in a general (German) population [1, 6]. Although a comparison of prevalence rates of BDD and muscle dysmorphia would require a structures diagnostic interview to ensure the diagnosis, especially since preoccupations about the own appearance are relatively common in the community [1], the results might indicate the population of military recruits as a population with higher prevalences. Our finding is comparable to the 13% self-reported prevalence among males enlisted in the U.S. military [10]. It is important to note that we exclusively used self-reported data based on the FSK as screening tool in order to identify individuals who might suffer from BDD symptoms instead of conducting diagnostic assessments according to the ICD or the DSM. This may have led to an over- or underestimation of BDD diagnosis in our results - although the specificity and sensitivity values reported herein were high based on the German validation study of the FSK. Additionally, since BDD is a shame-related disorder, anonymized self-assessment tools may also be helpful for obtaining honest responses – perhaps even more honest than the responses obtained from personal clinical interviews. However, future studies should include structured clinical interviews and ICD- or DSM-based diagnostical assessments to further investigate the prevalence of BDD in recruits. As expected, based on earlier studies (e.g., [8, 10]), the main area of preoccupation, in recruits showing BDD-symptoms, was their muscles. Contrary to some previous findings [8], the second most named area of preoccupation was their own genitals, as reported by 33.3% of the recruits. In the largest study to date looking at gender similarities and differences in 137 women and 63 men, Philips, Menard and Fay [8] found that only 17,5% of the male individuals named their genitals as the area of preoccupation. It would be interesting to further analyze whether age has an impact on the areas of preoccupation, as the sample of this study is substantially younger (M = 20.12 years, SD = 1.09, range: 18–24) than the 63 participants (M = 35.7 years, SD = 11.2) examined in Philips, Menard and Fays’ study [8]. Conversely, in our sample, the recruits reported less preoccupation with their hair (16.7%) than those in the study by Philips, Menards and Fays [8]. Again, age might have an impact, as getting bald might be an issue slightly later in life.

According to previous finding, a positive correlation between symptoms of BDD and depressive symptoms was expected, although the here found magnitude of the association was smaller than in previous research [3]. Nevertheless, these results are alarming, as recruits are expected to be mentally, intellectually and physically fit for service and for war. Forty-four recruits (35.5%) described hazardous or harmful alcohol use, which should be taken seriously. In regards to alcohol and/or drug abuse, no differences were found between those who were below or above the threshold for probable BDD. This is an unexpected result, as Gunstad and Philips [3] found high rates of substance abuse in individuals suffering from BDD. However, the rates of alcohol and drug abuse were generally so high throughout the sample that differences between clinical and nonclinical screened groups might have been too small. As in this sample the internal consistency of the AUDIT was only acceptable, these results need to be interpreted with caution. Future studies could also assess other common comorbidities such as other obsessive-compulsive spectrum disorders or social anxiety disorder for the overall picture.

Given our results showing the estimated prevalences of probable BDD, it is important to discuss which generalizations can be derived of our data. The clustered sample represents the full participation of a military unit and therefore can be assumed as representative for Swiss military recruits. Therefore, it bears the question to what extent our results are representative for young (Swiss) men in general? Approximately 2/3 of men are obliged to carry out military service in Switzerland, with the exception of those who have health issues (all recruits have to pass a test of physical and mental health before they begin their military service) and those who prefer social services as a substitute path. This results in approximately half of the Swiss male population, ca 20 years old, become a recruit. Thus, the sample represents a clustered sample of 50% of young Swiss men who are deemed physically and mentally fit and whose physical fitness is most likely above average (e.g., they have the ability to march). It is an open question whether symptoms of BDD would be more or less prevalent in an unselected sample of young men, i.e., especially in those with health concerns. Therefore, future studies could include a male age-controlled control group, to focus on differences in the setting and to further analyze whether general body dissatisfaction and/or BDD is more common in military recruits or not.

Referring to the setting of military services for which our results seem to be a reliable estimation, several implications have to be taken into account. Given that approximately one out of ten recruits shows symptoms of BDD and given that BDD typically occurs during adolescence, it appears vital to screen candidates for BDD symptoms before they begin military service. It seems also essential to increase awareness in recruits about BDD (e.g., with information materials) and to train military doctors, specifically with respect to screening BDD, regarding available information/psychoeducation, firstline treatments, and how to engage patients in treatment for BDD. This may be an important avenue to improve detection of BDD symptoms and secure access to treatment for young men suffering from symptoms of BDD. From a research perspective, the development of short and feasible screening tools with proven psychometric characteristics can help facilitate this task.

## Conclusion

Symptoms of BDD are common among Swiss military recruits, with their muscles being the main area of preoccupation, and showing a positive correlation with depressive symptoms. Further research is required and the design of the study could be improved upon (e.g., implementing diagnostic interviews). The results point towards the importance of building up an increased awareness of BDD among military recruits and leaders.

## Data Availability

The datasets generated and/or analyzed during the current study are not publicly available due to confidentiality, but are available on reasonable request from the corresponding author. The set of questionnaires used in this study only included published questionnaires (FKS [11], ADS [12], AUDIT [14], One-Question Screening for Drug Abuse [15], see methods section for details). As there was no German version of the One-Question-Screening [15], we translated it into German.

## Abbreviations

ADS: Allgemeine Depressionsskala
AUDIT: Alcohol Use Disorders Identification Unit
BDD: Body Dysmorphic Disorder
DSM: Diagnostic and Statistical Manual of Mental Disorders
FKS: Fragebogen körperdysmorpher Störungen
ICD: International Statistical Classification of Diseases and Related Health Problems
OCD: Obsessive Compulsive Disorder

## Footnotes

1 Regarding the specificity of the FKS, Buhlmann et al. [9] reports a specificity of 0.07. This is due to a printing error and was corrected to 0.93 (personal communication with Ulrike Buhlmann, July 2020).
